# Genetic and immune features of resectable malignant brainstem gliomas

**DOI:** 10.18632/oncotarget.19653

**Published:** 2017-07-28

**Authors:** Yang Zhang, Changcun Pan, Junmei Wang, Jingli Cao, Yuhan Liu, Yajie Wang, Liwei Zhang

**Affiliations:** ^1^ Department of Neurosurgery/China National Clinical Research Center for Neurological Diseases, Beijing Tiantan Hospital, Capital Medical University, Beijing 100050, China; ^2^ Department of Pathology, Beijing Neurosurgical Institute, Capital Medical University, Beijing 100050, China; ^3^ Core Laboratory for Clinical Medical Research, Beijing Tiantan Hospital, Capital Medical University, Beijing 100050, China

**Keywords:** brainstem gliomas, genetic features, programmed death ligand 1, CD8+ T cell infiltration, prognosis

## Abstract

We surveyed common genetic mutations (IDH1, H3F3A, PPM1D, and TP53) and immune features (PD-L1 expression and CD8^+^ T cell tumor infiltration) in a series of 62 malignant brainstem gliomas that were resected via microsurgery. IDH1 mutations were mutually exclusive with H3F3A mutations. IDH1 mutations appeared only in adults and occurred more frequently in tumors larger than 10cm^3^ (8/29 vs 1/32, Fisher’s exact test, p=0.010). H3F3A mutations occurred more frequently in children and adolescent patients (19/24 vs 18/38, chi-square test, p=0.013), low preoperative Karnofsky Performance Scale (KPS) patients (10/11 vs 20/43, chi-square test, p=0.021), and higher grade brainstem gliomas (8/21 in grade II vs 16/24 in grade III vs 13/17 in grade IV; chi-square test, p=0.038). PPM1D mutations clustered in H3F3A-mutated tumors (12/37), whereas were rare in H3F3A wildtype tumors (1/25). MGMT promoter methylations clustered in IDH1-mutated tumors (4/9), but were rare in H3F3A-mutated tumors (1/37). PD-L1 staining was detected in 59.7% of brainstem glioma specimens (37/62). High intra-tumoral CD8^+^ T cell density was less frequent in the H3F3A-mutated than H3F3A-wild-type tumors (4/37 vs. 11/25, p=0.005). Patients with H3F3A-mutated tumors (13.8 months overall survival) had much worse prognoses than those with IDH1-mutated (54.9 months, p=0.001) or H3F3A-IDH1 co-wildtype tumors (38.4 months, p=0.001). H3F3A mutations independently increased the relative risk of death as much as 4.19-fold according to a multivariate Cox regression model. Taken together, resectable malignant brainstem gliomas can be classified into three subtypes: H3F3A-mutated, IDH1 mutated and H3F3A-IDH1 co-wildtype tumors, which have distinct clinical characteristics, prognoses, genetic and immune features.

## INTRODUCTION

Brainstem gliomas occur in the midbrain, pons, and medulla oblongata. These tumors account for 1 to 2% of all adult primary brain tumors [1] and up to 20% of pediatric primary brain tumors [2]. The majority of brainstem gliomas are diffuse and intrinsic within the brainstem and are inaccessible by operation. Among these unresectable brainstem gliomas, diffuse intrinsic pontine gliomas (DIPGs), which diffusely occupy the pons, draw widespread attention from researchers in neuro-oncology, because DIPGs are the most common type of brainstem gliomas and the leading cause of brain-tumor-related death in children [3]. Biopsy for pathology plus radiotherapy is the standard care for this disease [3]. Considering the limited efficacy of current standard therapy and almost uniformly dismal prognosis, significant effort has been focused toward basic research and clinical trials for new therapies for this devastating disease [4, 5].

Other brainstem gliomas, which appear more focal or exophytic on imaging compared with classic DIPGs, are accessible by microsurgery [6, 7]. There are fewer studies on these resectable brainstem gliomas than on DIPGs [6]. In general, resectable brainstem gliomas are more heterogeneous than DIPGs with varying pathological types and variable prognoses [7]. Our previous whole exome sequencing study revealed that the most significant mutations in this group of brainstem gliomas occur in IDH1, H3F3A (H3.3), TP53, and PPM1D [8]. IDH1 and H3.3 mutations were mutually exclusive, and IDH1-mutated samples showed DNA methylation patterns distinct from H3.3-mutated samples[8]. These findings indicate that resectable brainstem gliomas can be classified into distinct subtypes according to their mutations in IDH1 and H3.3. PPM1D and TP53 mutations were another mutually exclusive pair of mutations in the resectable brainstem gliomas [8]. PPM1D mutations enhanced the ability of PPM1D to attenuate p53 activation by suppressing the activation of the DNA damage response checkpoint protein CHK2 [8]. These findings demonstrate that PPM1D and TP53 mutations together form the genetic basis for radiation-resistance.

However, in our previous study, only 33 cases of brainstem gliomas were included [8], the clinical characteristics of IDH1 or H3.3-mutated subtypes were not described, and overall survival rates were not significantly different for individuals with H3.3-mutated and IDH1-mutated brainstem gliomas. Additionally, O-methylguanine-DNA methyltransferase (MGMT) promoter methylation predicts better outcomes for patients who receive TMZ-based chemotherapy for supratentorial gliomas [9]. There was no report regarding to the prevalence of MGMT promoter methylation in brainstem gliomas, except one study reporting that positive MGMT staining by immunohistochemistry (correlate with unmethylated MGMT promoter) was found in 64.7% of adult brainstem gliomas [10].

In general, the prognosis of resectable brainstem gliomas is worse than their supratentorial counterparts, due to a more difficult operation and less effective radiotherapy and standard chemotherapy [11, 12]. Immunotherapy offers new opportunities to develop treatments for glioma patients [13]. A new type of immunotherapy against programmed death 1 (PD-1) and its ligand, PD-L1, has exhibited impressive anti-tumor activity in several cancers [14–18]. PD-1 and PD-L1 are a pair of immune checkpoint molecules that constitute a major immune resistance mechanism within the tumor microenvironment [19, 20]. Success of an anti-PD-1/PD-L1 therapy requires the expression of PD-L1 on tumor cells and the presence of high infiltrative densities by CD8^+^ T cells [21–25]. A recent study reported high levels of PD-L1 expression and increased CD8^+^ T cell infiltration within glioblastomas (GBMs), most of which are located supratentorially [26]. These results imply the availability of immune checkpoint therapy for GBM patients. Therefore, several phase I/II clinical studies are ongoing to determine the efficacy and safety of anti-PD-1/PD-L1 therapies to treat GBMs and DIPGs [27]. However, the patterns of PD-L1 expression and CD8^+^ T cell infiltration within resectable brainstem gliomas, which have a unique molecular pathogenesis compared with supratentorial gliomas [8, 28], remain unclear.

Here, we surveyed the genetic mutations in IDH1, H3.3, PPM1D, and TP53 in a series of 62 brainstem gliomas that were resected via microsurgery. MGMT promoter methylations were also determined. The PD-L1 expression and CD8^+^ T cells infiltration were evaluated as two immune features in the study, because they are major participants within tumor microenvironment, and more importantly, they are predictors for better outcomes of anti-PD-1/PD-L1 therapy [21–25].

## RESULTS

### Clinical characteristics

Patient characteristics, including baseline clinical parameters, tumor size and location, pathological diagnosis, extent of resection and postoperative therapy modalities, are summarized in Table 1 . The ratio of males to females was 38 to 24. The median age at diagnosis was 23 years old (range 2-59 years). Both pediatric and adult brainstem gliomas were included in the study. There were two incidence age peaks: 5-9 years and 20-44 years. The first age peak included 21% (13/62) of the studied patients and the second included 50% (31/62) patients ( Supplementary Figure 1). The median Karnofsky Performance Score (KPS) before operation was 70 (range 50-100). The most common site for the brainstem gliomas was the pons (36, 58.1%), followed by the medulla (18, 29.0%). Total or subtotal resection was achieved in 74.2% of the patients. The proportions of tumors that were astrocytic gliomas, oligodendroglial gliomas, and GBMs were 48.1%, 22.2% and 29.6%, respectively. According to WHO grading, 33.9% (21), 38.7% (24) and 27.4% (17) of the included cases were determined as grade II, grade III and grade IV gliomas. A total of 25 cases (40.3%) received TMZ-based chemoradiotherapy after operation, 11 cases received single radiotherapy, 1 received single TMZ chemotherapy and 1 received an immunotherapy. The median overall survival time was 12.4 months (range 0.5 to 71.5 months).

**Table 1:** Clinical characteristics of the included patients

	Number	Proportion (%)
**Gender:**		
Male	38	61.3
Female	24	38.7
**Median age at diagnosis, years (range):**	23 (2-59)	
**Median preoperative KPS (range):**	70 (50-100)	
**Tumor locations:**		
Medulla	18	29.0
Pons	36	58.1
Midbrain	6	9.7
Multiple	2	3.2
**Median tumor size, cm**^3^ **(range):**	9.7 (1.0-40.6)	
**Resection extent:**		
Total or Subtotal	46	74.2
Partial	14	22.6
Unknown	2	3.2
**Pathological type:**		
A or AA	30	48.4
O or AO	15	24.2
GBM	17	27.4
**Pathological grade (WHO):**		
II	21	33.9
III	24	38.7
IV	17	27.4
**Postoperative first-line therapy:**		
Radiotherapy	11	17.7
Chemotherapy	1	1.6
Chemoradiotherapy	25	40.3
No tumor specific therapy	21	33.9
Other	1	1.6
Unknown	3	4.8
**Status at last follow-up:**		
Alive	23	37.1
Dead	35	56.5
Unknown	4	6.5
**Median overall survival months (Range):**	12.5 (0.5-71.5)	

A: astrocytoma, AA: anaplastic astrocytoma, O: oligodendroglioma/oligoastrocytoma, AO: anaplastic oligodendroglioma/anaplastic oligoastrocytoma, KPS: Karnofsky Performance Score.

### Genetic features

**Figure 1:**
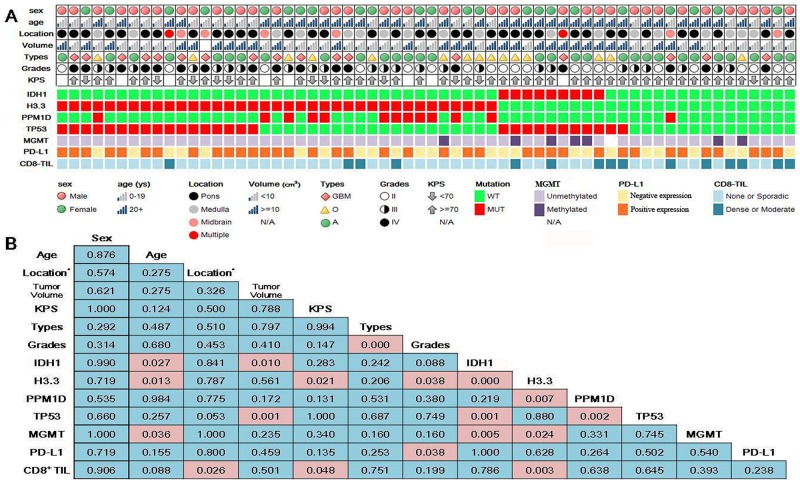
Clinical characteristics and genetic and immune features of resectable brainstem gliomas **(A)** Overview of the clinical characteristics, genetic and immune features. **(B)** P values from correlative analyses among the clinical characteristics, genetic and immune features; *Location: pons and non-pons.

We surveyed mutations in IDH1, H3.3, PPM1D and TP53 and methylation status at MGMT promoter for all cases. As shown in Figure 1A, H3.3 mutations (37, 59.7%) are the most frequent in the brainstem gliomas, followed by TP53 (28, 45.2%), PPM1D (13, 21.0%) and IDH1 (9, 14.5%) mutations. MGMT promoter methylation was detected in 7 cases among 61 patients (undetermined in one case). As we reported previously [25], IDH1 mutations were mutually exclusive with H3.3 mutations (Figure 1, Fisher’s exact test, p<0.001), and TP53 mutations were also mutually exclusive with PPM1D mutations except for one case of co-mutations (Figure 1, Fisher’s exact test, p=0.002). PPM1D was mutated in 12 of 37 H3.3-mutated cases compared to only one case of PPM1D mutation in 25 H3.3-wildtype cases (chi-square test, p=0.007). IDH1 mutations were only present in TP53-mutated cases (Figure 1, Fisher’s exact test, p<0.001). MGMT promoter methylation was rarer in H3.3-mutated cases (1/37) than in H3.3-wild-type cases (6/24, chi-square test, p=0.024), and more frequent in IDH1-mutated cases (4/9) than in IDH1-wildtype cases (3/52, chi-square test, p=0.005).

Genetic mutations were quite similar within the group from patients 0-19 years of age or within those from patients >=20 years, but rather different between the two groups ( Supplementary Table 1). Therefore, patients 0-19 years and >=20 years were grouped as the children/adolescent and adult groups, respectively. As shown in Figure 1, IDH1 mutations were absent in children/adolescent patients (0-19 years) and only occurred in adult patients (>=20 years) (chi-square test, p=0.027). In contrast, H3.3 mutations occurred more frequently in children/adolescent patients (19/24) compared to adult patients (18/38, chi-square test, p=0.013). Similar to IDH1 mutations, MGMT promoter methylation was also absent in children/adolescent patients and only occurred in adult patients (7/37) (Fisher’s exact test, p=0.036). IDH1 (8/29 vs 1/32, Fisher’s exact test, p=0.010) and TP53 mutations (19/29 vs 8/32, chi-square test, p=0.001) occurred more frequently in larger volume tumor (>10cm^3^). Nearly all (10/11) patients with low KPS (<70) before operation have H3.3 mutations in their tumors, whereas 20 of 43 patients with KPS>=70 had H3.3-mutated tumors (chi-square test, p=0.021). The proportions of H3.3 mutations also correlated with WHO malignancy grades with 38.1% (8/21) in grade II, 66.7% (16/24) in grade III and 76.5% (13/17) in grade IV (chi-square test, p=0.038).

### Immune features

**Figure 2:**
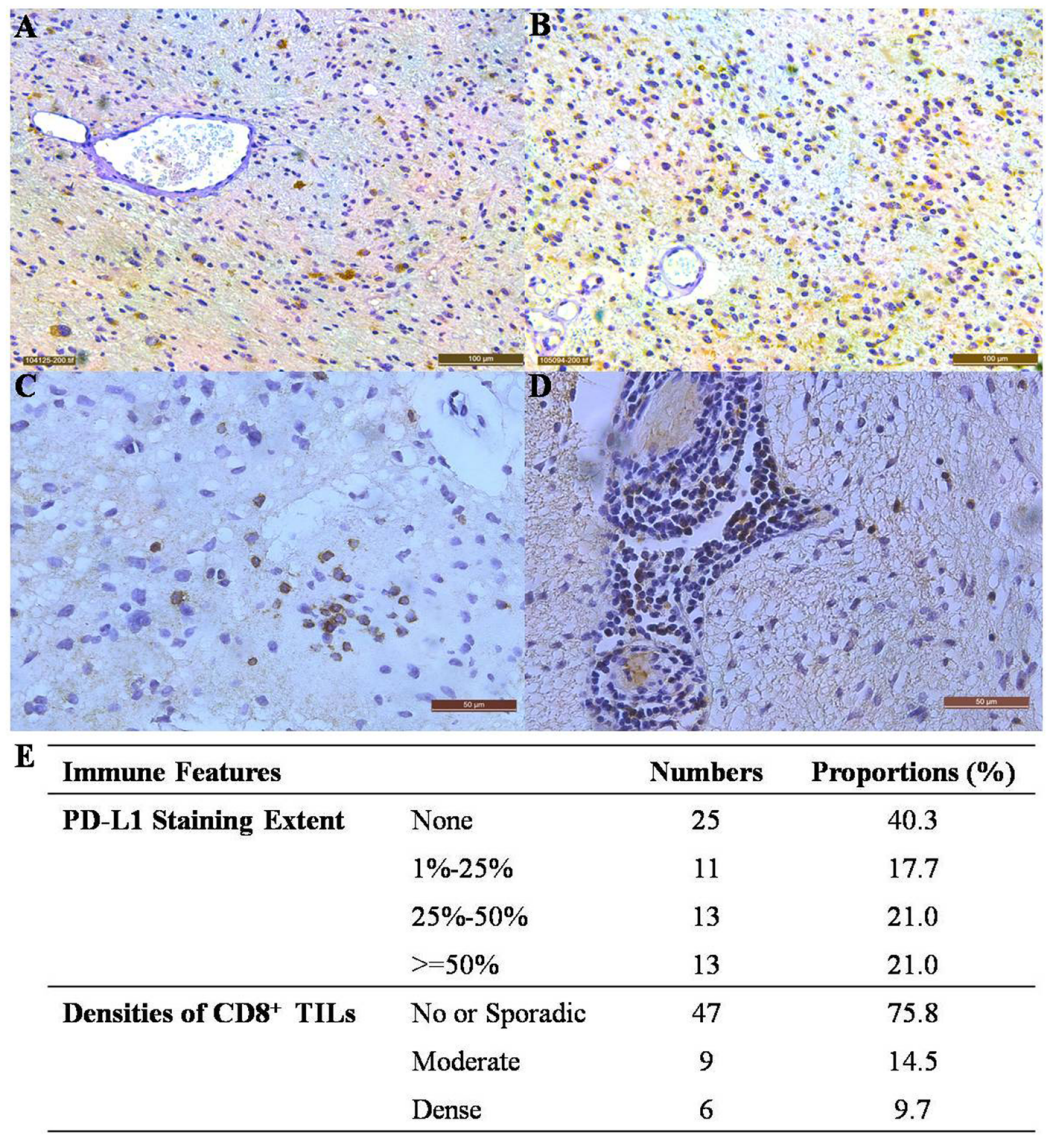
Immune features of resectable brainstem gliomas **(A)** PD-L1 staining extent = 15% (200× magnification). **(B)** PD-L1 staining extent = 75% (200× magnification). **(C)** Moderate infiltration by CD8^+^ T cells within the tumor (400× magnification). **(D)** Dense infiltration by CD8^+^ T cells (200× magnification). **(E)** The extent of PD-L1 staining and infiltrative densities of CD8^+^ T cells in resectable brainstem gliomas.

The expression of PD-L1 and infiltration of CD8^+^ T cells were used as two immune features for evaluating the immune microenvironments within brainstem gliomas. The extent of PD-L1 staining varied in brainstem gliomas (Figure 2A–2B). More than a half of the brainstem gliomas (37 out of 62 cases) expressed varying amounts of PD-L1 (Figure 2E); 11 cases showed staining in less than 25% of the tissue, 13 showed staining in 25 to 50% of the tissue, and 13 showed staining in greater than 50% of the tissue (Figure 2E). The dominant pattern of PD-L1 staining was cytoplasmic and typically disperse with no enhancement of the vascular structure throughout the tumor tissue (Figure 2A–2B). Moderate or dense infiltration by CD8^+^ T cells (considered as high intratumoral CD8^+^ T cell density for further analyses) was observed in 15 of the brainstem gliomas (Figure 2C–2E).

To validate semi-quantitative results from the IHC tests, we quantified the transcriptional level of PD-L1 within 29 frozen tumor tissue specimens from the included patients. As shown in Supplementary Figure 2, the transcriptional level of PD-L1 was significantly higher in the group of positive PD-L1 staining than the negative PD-L1 staining group (student’s t-test, p=0.045). There was no significant difference of PD-L1 transcriptional levels among the groups with different PD-L1 staining extents. Therefore, samples were divided into negative and positive PD-L1 expression groups for further analyses (Figure 1).

As shown in Figure 1B, the immune features correlated with the genetic features and clinical characteristics of brainstem gliomas. The prevalence of positive PD-L1 staining was 76.5% (13/17) in grade IV and 66.7% (16/24) in grade III, which was significantly higher than 38.1% (8/21) in grade II brainstem gliomas (chi-square test, p=0.038). High intra-tumoral CD8^+^ T cell density was rarer in pontine gliomas (chi-square test, p=0.026) and H3.3-mutated cases (chi-square test, p=0.003). Interestingly, in contrast to H3.3 mutations, no cases with low KPS (<70) before operation showed high intra-tumoral CD8^+^ T cell density, whereas 14 out of 43 cases with KPS more than 70 had dense or moderate CD8^+^ T cell infiltration within tumors (Fisher’s exact test, p=0.048).

### Prognostic factors

**Figure 3:**
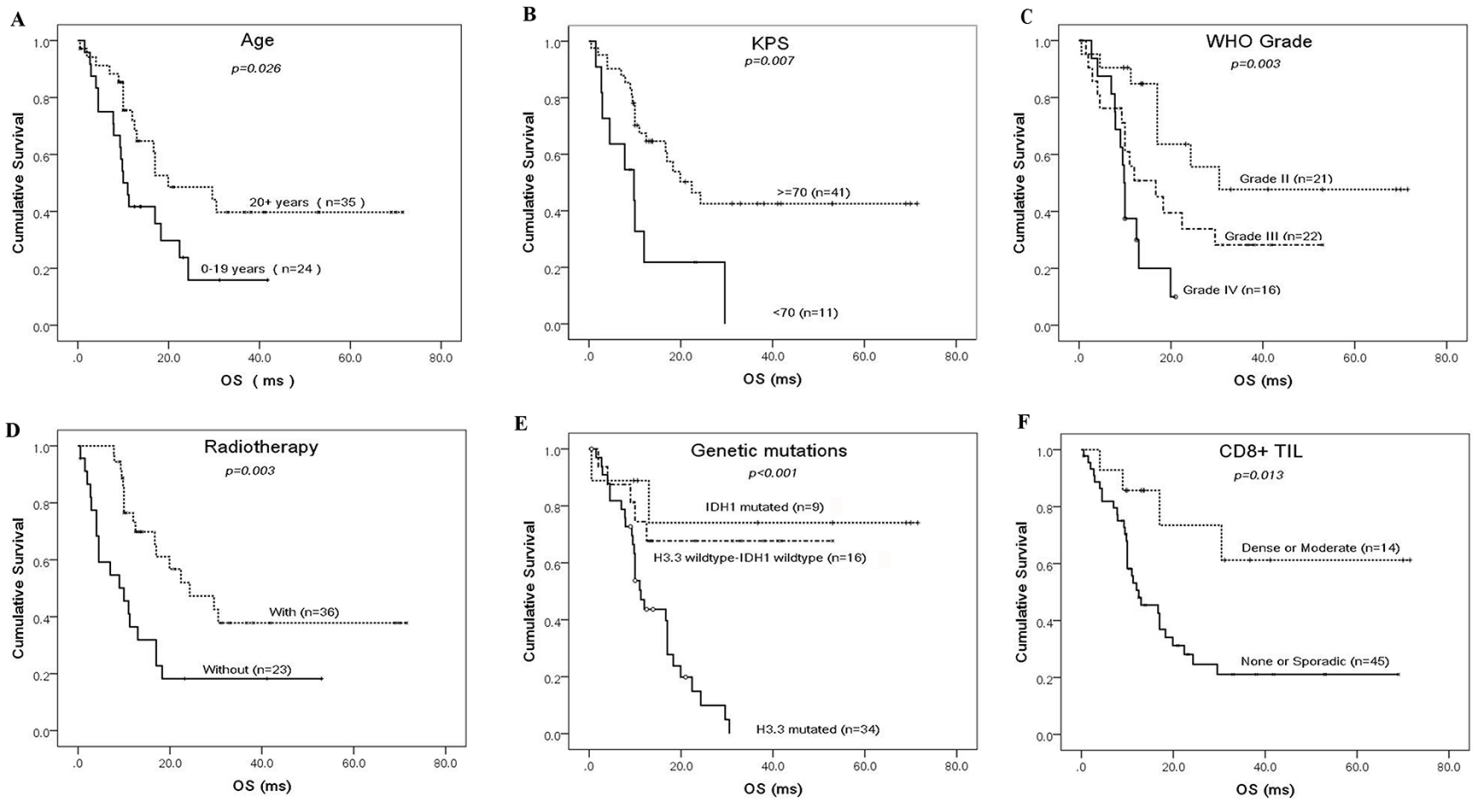
Prognostic factors for the overall survival of resectable brainstem glioma patients Kaplan-Meier curves for the cohort of brainstem glioma patients according to age **(A)**, preoperative KPS **(B)**, WHO malignancy grades **(C)**, receiving radiotherapy **(D)**, genetic mutations **(E)** and density of CD8^+^ TIL (tumor infiltrative lymphocyte) **(F)**.

The median overall survival time was 12.5 months in all the included patients (Table 1). The factors of patients’ age, KPS level before operation, WHO malignancy grade, receiving radiotherapy after operation, genetic mutations in IDH1 and H3.3, and high density of CD8^+^ T cell infiltration, significantly affect the overall survival of brainstem glioma patients (Figure 3). Patients <20 years of age had a shorter median overall survival than those >=20 years (10.0 months vs 19.9 months, p=0.026). A KPS < 70 before operation indicated worse prognosis than KPS >=70 (9.8 months vs 22.4 months, p=0.007). Median overall survival became shorter with the increasing the WHO malignancy grades (30.5 months in grade II vs 16.7 months in grade III vs 9.8 months in grade IV, p=0.003). Postoperative radiotherapy significantly improved overall survival (24.3 months with radiotherapy vs 10.0 months without radiotherapy, p=0.003). H3.3 mutations indicated much worse prognosis than IDH1 mutations (13.8 months vs 54.9 months, p=0.001) or H3.3-IDH1 co-wild-type (13.8 months vs 38.4 months, p=0.001). High CD8^+^ T cell density within the tumor microenvironment was a prognostic indicator for favorable outcomes in patients with brainstem gliomas (50.5 months vs 23.9 months, p=0.013).

A multivariate Cox regression model was fitted to identify independent prognosticators for the overall survival of brainstem glioma patients. As shown in Table 2 , receiving postoperative radiotherapy was the strongest prognostic indicator and reduced the relative risk of death by as much as 6.25-fold. H3.3 mutations increased the relative risk of death as much as 4.19 fold. Higher WHO grade and low KPS before operation (<70) were also independent factors that predicted dismal prognosis for brainstem glioma patients, with the relative risk of 2.97 and 2.52, respectively.

**Table 2:** Cox regression model predicting median overall survival in resectable brainstem glioma patients

Parameters		Hazard ratio	95% CI	p
H3.3:	Mutated vs. Wild-type	4.19	1.42-12.35	0.009
Grade:	IV vs. III vs. II	2.97	1.42-6.24	0.004
KPS:	<70 vs.>=70	2.52	1.03-6.15	0.042
Radiotherapy:	With vs. Without	0.16	0.06-0.38	<0.001

## DISCUSSION

In this study, we reported genetic and immune features of one of the largest series of resectable brainstem gliomas. We sequenced mutations of IDH1, H3.3, PPM1D and TP53, because they are the most significant genetic mutations of resectable brainstem gliomas, as we reported previously [8]. IDH1 mutations are the most frequent mutations in supratentorial gliomas [29], but were not as frequent in the brainstem gliomas (9, 14.5%). In contrast, H3.3 mutations (37, 59.7%) prevailed in these cases, because H3.3 mutations are feature mutations in midline gliomas including brainstem gliomas [30]. Consistent with our previous study on resectable brainstem gliomas [8], IDH1 and H3.3 mutations were mutually exclusive (Figure 1). According to DNA methylation patterns, IDH1 mutations lead to hypermethylation throughout the genome, H3.3 mutations were associated with hypomethylation, and IDH1-H3.3 co-wild-type tumors showed an intermediate phenotype [8]. All these findings implied that resectable brainstem gliomas can be stratified into three subtypes: H3.3-mutated, IDH1-mutated, and IDH1-H3.3 co-wildtype.

Clinical characteristics and prognosis differed substantially among these three subtypes. IDH1 mutations only appeared in adult group (20+ years) but were absent in children/adolescent group (0-19 years)(chi-square test, p=0.027). In contrast, H3.3 mutations were more common in children/adolescent patients than in adult patients (79.2% vs 47.4%, chi-square test, p=0.013). IDH1 mutations occurred more frequently in tumors >10cm^3^ in volume (Fisher’s exact test, p=0.010), and IDH1-mutated tumors appeared larger than IDH1-wildtype tumors (18.5 cm^3^vs. 11.2 cm^3^, student’s t-test, p=0.035). H3.3-mutated tumors occurred more frequently in patients with KPS<70 (chi-square test, p=0.021) and were more likely to be high-grade tumors (chi-square test, p=0.038). More importantly, H3.3-mutated tumors exhibited much worse prognosis than IDH1-mutated (13.8 months vs. 54.9 months, p=0.001) or H3.3-IDH1 co-wild-type tumors (13.8 months vs. 38.4 months, p=0.001) (Figure 3). H3.3 mutations increased the relative risk of death as much as 4.19 fold according to a multivariate Cox regression model (Table 2).

Yamasaki et.al reported that a patient age <20 years was one of the poor prognostic factors in a retrospective study of 23 patients with diffuse brainstem gliomas [31]. Lachi et.al reported a similar result that pediatric age group (<15 years) was associated with worse prognosis than in adults (>=15 years) in a series of 48 patients with brainstem gliomas treated by radiation therapy [32]. Consistent with these previous studies, we showed that the children/adolescent group (0-19 years) had a shorter median overall survival than adults (20+ years) (10.0 months vs. 19.9 months, p=0.026, Figure 3). The worse prognosis in the children/adolescent group is attributed to the absence of IDH1 mutations and clustering of H3.3 mutations (Figure 1).

**Figure 4:**
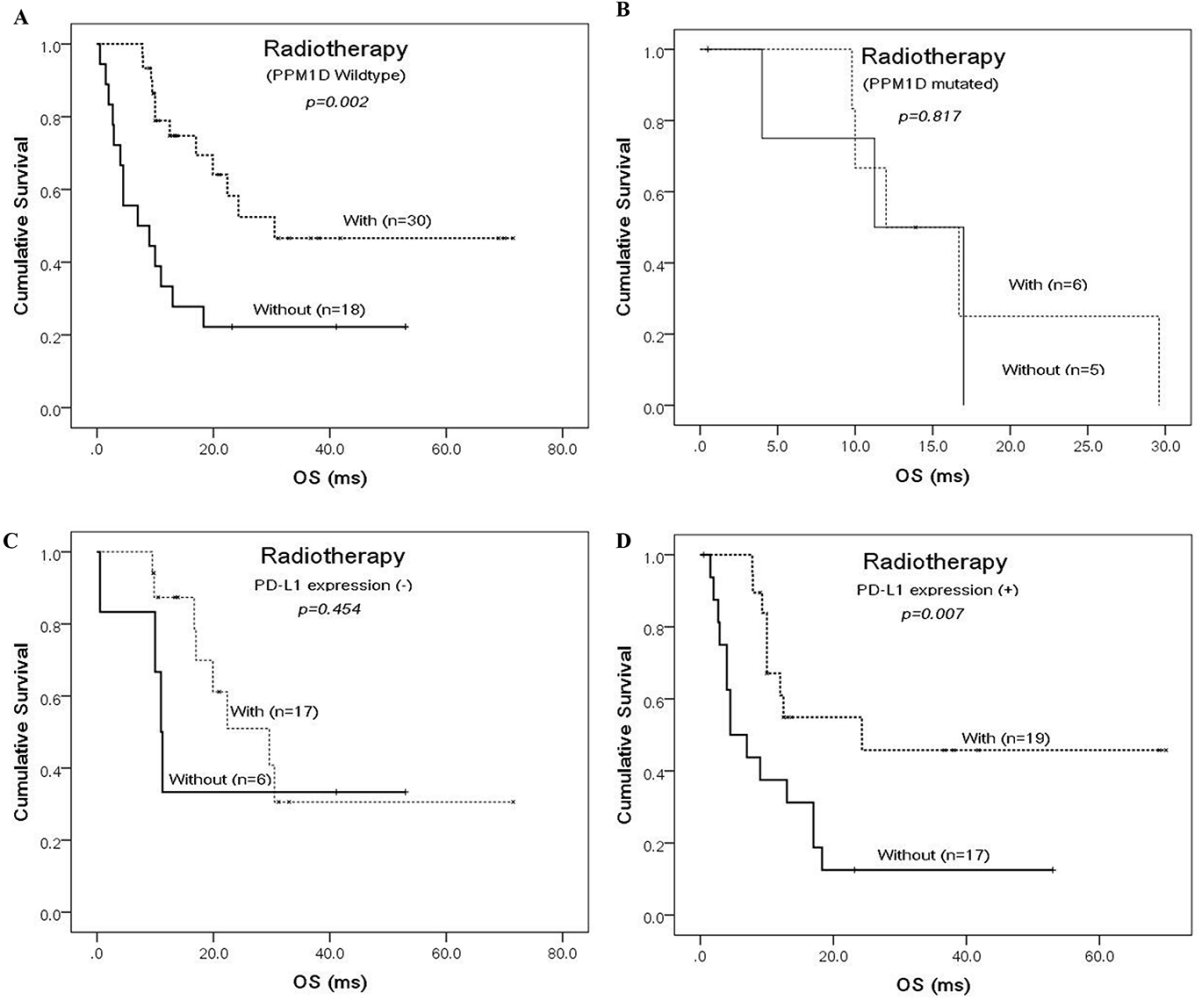
Prognostic factors that predict whether brainstem glioma patients benefit from radiotherapy or not Kaplan-Meier curves according to receiving radiotherapy for the cohort of brainstem glioma patients, which were classified as PPM1D-wildtype **(A)** and mutated **(B)** group or PD-L1 expression negative **(C)** and positive **(D)** group.

Radiotherapy is still the standard care of treatment for brainstem gliomas [3, 6]. In this series of cases, radiotherapy significantly affected the overall survival of brainstem glioma patients who underwent prior microsurgery (Figure 3). It was the strongest prognosticator and reduced the relative risk of death by as much as 6.25-fold (Table 2). PPM1D mutations clustered in H3.3-mutated subtype (12/37 vs 1/25, chi-square test, p=0.007) whereas TP53 mutations were rare in H3.3-IDH1 co-wild-type subtype (2/16 vs 26/46, chi-square test, p=0.002). They were another mutually-exclusive pair of mutations that together formed the genetic basis for radiation-resistance in brainstem gliomas [8]. However, there has been no report on whether they can finally affect the radiotherapy outcome of brainstem glioma patients. As shown in Figure 4, radiotherapy did not improve the patients’ outcome when PPM1D was in mutation. But we did not observe the same affect when TP53 was mutated. Therefore, PPM1D mutations predicted the limited benefits of radiotherapy on brainstem glioma patients. It warrants new therapeutic modalities for PPM1D-mutated patients [25, 33].

MGMT promoter methylation predicts better outcome for patients who receive TMZ-based chemotherapy for supratentorial gliomas [9]. A previous study found positive MGMT staining (correlates with an unmethylated MGMT promoter) by the IHC method in 64.7% of adult brainstem gliomas [10]. In this study, we identified only 7 cases with MGMT promoter methylations. Partly due to its low prevalence in brainstem gliomas (7/61), TMZ-based chemotherapy did not improve the overall survival in this series of patients (Log-Rank test, p=0.173) and success was limited in previous studies [6, 34, 35]. Importantly, MGMT promoter methylation was unevenly distributed between the different groups of brainstem gliomas. For example, it occurred solely in adult group (7/37) (>= 20 years) but not in children/adolescent patients (0-19 years). It clustered in IDH1-mutated subtype (4/9) compared with the rarity in H3.3-mutated subtype (1/37) . Therefore, TMZ-based chemotherapy would be effective in adult brainstem gliomas, especially for the adult patients with IDH1-mutated tumors. And future clinical trials of TMZ based chemotherapy would be better on these patients.

Considering the generally dismal prognosis of brainstem glioma patients [11], it is necessary to accelerate the translation of new therapies, such as immunotherapy. Strategies aimed at blocking PD-1-PD-L1 ligation are promising immunotherapies for many cancers [36]. Although controversial, responses to anti-PD-1/PD-L1 therapies are dependent on the expression of PD-L1 [21]. In this study, we detected a high prevalence (37/62, 59.7%) of PD-L1 staining in brainstem glioma specimens (Figure 2E). Our real-time PCR result confirmed the difference of specimens with positive and negative PD-L1 staining at the RNA level ( Supplementary Figure 2). The high prevalence provides a rationale for the initiation of clinical trials aimed at determining the efficacy of anti-PD-1/PD-L1 therapies for this deadly type of brain tumor. An clinical trial of an anti-PD-1 therapy in DIPGs is currently being conducted (Pidilizumab, NCT01952769). The results in this study of resectable brainstem gliomas support the expansion of therapies for this indication. PD-L1 expression was also correlated with malignancy grades in brainstem gliomas, with the proportions of positive PD-L1 staining highest in grade IV gliomas. Yao Y et al. observed the same correlation using Western blot analysis, indicating that high-grade gliomas expressed more PD-L1 than low-grade gliomas [37]. Interestingly, similar to PPM1D mutations, the state of PD-L1 expression seemed to predict whether the patients would benefit from receiving radiations after operation (Figure 4). The underlying mechanisms of these correlations are still unknown and warrants for further exploration.

High densities of infiltrating CD8^+^ T cells within tumors, which usually indicates an active antitumor immune response, are also correlated with response to anti-PD-1/PD-L1 therapy [25]. In this group of brainstem gliomas, 15 out of 62 (24.2%) cases showed moderate or dense infiltration (Figure 2C). High density infiltration by CD8^+^ T cell was more frequent in non-pontine gliomas (chi-square test, p=0.026). As shown in Figure 1, H3.3-mutated tumors exhibits less CD8^+^ T cell infiltration (4/37) than the H3.3-wildtype (11/25) (chi-square test, p=0.003). It implied that anti-tumor response was smaller for H3.3-mutated brainstem gliomas than the wildtype. High infiltration of CD8^+^ T cell correlated with higher KPS before operation (14/43 in KPS>=70 vs 0/11 in KPS<70, Fisher’s exact test, p=0.048). It indicated that higher KPS was linked to better immune systems and then more anti-tumor response within brainstem gliomas. Consistent with previous studies in supratentorial gliomas [38, 39], a high infiltrative density of CD8^+^ T cells was correlated with optimistic outcomes in brainstem gliomas (Figure 3). This correlation can be explained by low prevalence of H3.3-muations and high KPS levels in the cases with high CD8^+^ T cells infiltration.

Due to the rarity and heterogeneity of resectable brainstem glioma [7], the low sample size in some subgroups limits the results and conclusions of the study. For example, we cannot confirm the effect of MGMT promoter methylation on chemotherapy outcome of brainstem glioma, because only seven cases with MGMT promoter methylation were in this study. The mutations in IDH usually predict better prognosis for supratentorial gliomas [29], but in this series of brainstem gliomas with nine cases of IDH1 mutations, the overall survival of IDH1-mutated subtype was not longer than IDH1-H3.3 co-wild-type subtype (Figure 3). So it is necessary to expand the current sample size in future studies for overcoming these limitations.

There is another limitation in this study. The link of PD-L1 expression to the efficacy of an anti-PD-1/PD-L1 therapy usually requires membranous expression of PD-L1 [21–25]. However, the major pattern of PD-L1 staining was cytoplasmic in this series of brainstem glioma specimens. Berghoff et al. also reported the cytoplasmic staining of PD-L1 in supratentorial GBM [26]. The cytoplasmic staining could be attributed to internalized surface PD-L1 molecules, as previously described in lymphoma [40]. Berghoff et al. described that membranous staining was often observed in epithelioid GBM cells [26]. The low prevalence of membranous staining in brainstem gliomas could be attributed to the uncommon epithelioid GBM cells observed in this group of cases. Although the presence of the PD-L1 target implied the availability of anti-PD-1/PD-L1 therapy for resectable brainstem gliomas, it is still unclear whether the cytoplasmic staining seen in this study would predict a similar response to anti-PD-1/PD-L1 therapy on those cancers with membranous expression of PD-L1. Concerning that the clinical significance of PD-L1 cytoplasmic staining is not well characterized, in comparison to other cancers, it is more essential to examine the association between the staining pattern and extent of PD-L1, and response rates in future clinical studies.

## MATERIALS AND METHODS

### Patients

A total of 62 patients with brainstem gliomas were retrospectively analyzed in this study. All of the included patients underwent microsurgical resection at Beijing Tiantan Hospital between 2009 and 2016. The associated clinical and pathologic information was extracted from clinical information databases. The tumor tissue specimens were obtained from the brain tumor biobank of Beijing Tiantan Hospital. This study was supported by the Neurosurgical Clinical Information and Biobanking Project of Beijing Tiantan Hospital (Brain Tumor Section) and approved by the ethics committee at Beijing Tiantan Hospital (KY2014-021-02).

### Genetic features evaluation

Formalin-fixed and paraffin-embedded (FFPE) patient samples were sequenced using the Sanger method to identify genetic mutations (IDH1, H3.3, PPM1D and TP53) common to resectable brainstem gliomas [8]. IDH1 mutation sequencing focused on the R132 mutation whereas H3.3 mutation sequencing focused on the K27 mutation. The sequencing of PPM1D and TP53 covered the known hotspot gene mutations, which were reported in resectable brainstem glioma [8]. MGMT promoter methylation states in these samples were determined by the pyrosequencing method. The mutation sequencing and analyses were performed by Beijing Genetron Health, Co. Ltd.

### Immune features evaluation

The immunohistochemical (IHC) methods were applied to evaluate the PD-L1 expression and CD8^+^ T cells infiltrative density within brainstem gliomas. Briefly, FFPE blocks were cut into serial 3 µm-thick slices. After deparaffinization and rehydration, the slides underwent heat-induced epitope recovery according to a routine procedure. The sections were blocked in a goat serum working solution for 1 hour and then were incubated overnight at 4°C in anti-PD-L1(Cat# ab205921, Abcam, Cambridge, UK) antibodies diluted 1:100 and anti-CD8 (Cat#ab4055, Abcam, Cambridge, UK) antibodies diluted 1:400 in a stock solution of 0.1% PBST and 10% goat serum. After the incubation in secondary antibody solutions for 1 hour, the sections were stained using a DAB peroxidase substrate solution for 20 minutes, and counterstained with hematoxylin for 15 minutes. Finally, the sections were dehydrated and mounted according to a standard protocol. PD-L1 expression was semi-quantified using the following criteria: “(i) no positive tumor area; (ii) expression in <25% of non-necrotic tumor areas; (iii) expression in >25% and <50% of non-necrotic areas; and (iv) expression in >50% of non-necrotic areas” [26]. The density of the CD8^+^ T cells was semi-quantified according to criteria described in previous studies [41] and graded as no or sparse, moderate and dense infiltration.

### Quantification of PD-L1 RNA by real-time qPCR

Among the included cases, 29 had fresh frozen tumor tissue specimens, which were used to quantify the transcriptional levels of PD-L1 with real-time quantitative reverse transcription PCR (RT-qPCR). RT-qPCR was performed and optimized as described previously [42]. Briefly, tissues were placed in liquid nitrogen and ground thoroughly with a mortar and pestle. Total RNA was isolated using TRIzol Reagent (Thermo Fisher, MA, USA) according to the manufacturer’s instructions. The quantity of RNA in each sample was assessed by a Nano Drop ND-1000 spectrophotometer (Thermo Fisher, MA, USA), while RNA integrity was determined by denaturing formaldehyde gel electrophoresis. The RNAs were dissolved in diethylpyrocarbonate (DEPC)-treated water and reverse transcribed by the SuperScript III First Strand Synthesis Super Mix Kit (Thermo Fisher, MA, USA) according to the manufacturer's instruction. cDNA was quantitated using a TaqMan-based Real-Time qPCR assay with TaqMan Universal Master Mix II (Thermo Fisher, MA, USA). The following primers and probe were synthesized by Synbio Tech (Suzhou, China): PD-L1 forward primer (5’-CCTGAGGAAAACCATACA -3’), PD-L1 reverse primer (5’-CACCAAGGCATAATAAGATG-3’), PD-L1 probe (FAM-ACTACCTCTGGCACATCCTCC-BHQ1), GAPDH forward primer (5’-GTGTGAACCATGAGAAGTA-3’), GAPDH reverse primer (5’-TCCACGATACCAAAGTTG-3’), GAPDH probe (HEX-CAACAGCCTCAAGATCATCAGCAA-BHQ1). Real-Time qPCR reactions were performed on 96-well plates and run in the CFX 96 system (Bio-Rad Laboratories, CA, USA). GAPDH was included as reference genes. All reactions were performed in triplicate. The relative expression was analyzed using Bio-Rad CFX Manager Software.

### Statistical analyses

All statistical analyses were performed using SPSS software version 20 (IBM, New York, USA). Categorical data were compared using Chi-squared test or Fisher’s exact test. Continuous data were compared using Student’s t-test. The Kaplan-Meier analysis was used to estimate the overall survival time from surgery to either death or last follow-up. The Log-Rank test was applied to estimate group differences and examine the factors that impact the overall survival of brainstem glioma patients. A Cox regression model was fitted to select the independent prognostic factors. A 2-tailed p-value<0.05 was considered to indicate significance.

## CONCLUSION

Resectable malignant brainstem gliomas can be classified into three subtypes: H3F3A-mutated, IDH1 mutated and H3F3A-IDH1 co-wildtype tumors, which have distinct clinical characteristics, prognoses, genetic and immune features. A rather high prevalence of PD-L1 staining was present in resectable brainstem gliomas.Receiving postoperative radiotherapy was the strongest factors that independently prognosticated the outcome of patients in this series. PPM1D mutations and PD-L1 expression appeared to predict whether the brainstem glioma patients would benefit from postoperative radiotherapy.

## SUPPLEMENTARY MATERIALS FIGURES AND TABLE



## References

[1] Ostrom QT, Gittleman H, Liao P, Rouse C, Chen Y, Dowling J, Wolinsky Y, Kruchko C, Barnholtz-Sloan J (2014). CBTRUS statistical report: primary brain and central nervous system tumors diagnosed in the United States in 2007-2011. Neuro Oncol.

[2] Smith MA, Freidlin B, Ries LA, Simon R (1998). Trends in reported incidence of primary malignant brain tumors in children in the United States. J Natl Cancer Inst.

[3] Vanan MI, Eisenstat DD (2015). DIPG in children - what can we learn from the past?. Front Oncol.

[4] Goodwin CR, Xu R, Iyer R, Sankey EW, Liu A, Abu-Bonsrah N, Sarabia-Estrada R, Frazier JL, Sciubba DM, Jallo GI (2016). Local delivery methods of therapeutic agents in the treatment of diffuse intrinsic brainstem gliomas. Clin Neurol Neurosurg.

[5] Johung TB, Monje M (2017). Diffuse intrinsic pontine glioma: new pathophysiological insights and emerging therapeutic targets. Curr Neuropharmacol.

[6] Hu J, Western S, Kesari S (2016). Brainstem glioma in adults. Front Oncol.

[7] Reyes-Botero G, Mokhtari K, Martin-Duverneuil N, Delattre JY, Laigle-Donadey F (2012). Adult brainstem gliomas. Oncologist.

[8] Zhang L, Chen LH, Wan H, Yang R, Wang Z, Feng J, Yang S, Jones S, Wang S, Zhou W, Zhu H, Killela PJ, Zhang J (2014). Exome sequencing identifies somatic gain-of-function PPM1D mutations in brainstem gliomas. Nat Genet.

[9] Ludwig K, Kornblum HI (2017). Molecular markers in glioma. J Neurooncol.

[10] Babu R, Kranz PG, Agarwal V, McLendon RE, Thomas S, Friedman AH, Bigner DD, Adamson C (2014). Malignant brainstem gliomas in adults: clinicopathological characteristics and prognostic factors. J Neurooncol.

[11] Reithmeier T, Kuzeawu A, Hentschel B, Loeffler M, Trippel M, Nikkhah G (2014). Retrospective analysis of 104 histologically proven adult brainstem gliomas: clinical symptoms, therapeutic approaches and prognostic factors. BMC Cancer.

[12] Eisenstat DD, Pollack IF, Demers A, Sapp MV, Lambert P, Weisfeld-Adams JD, Burger PC, Gilles F, Davis RL, Packer R, Boyett JM, Finlay JL (2015). Impact of tumor location and pathological discordance on survival of children with midline high-grade gliomas treated on Children's Cancer Group high-grade glioma study CCG-945. J Neurooncol.

[13] Reardon DA, Freeman G, Wu C, Chiocca EA, Wucherpfennig KW, Wen PY, Fritsch EF, Curry WT, Sampson JH, Dranoff G (2014). Immunotherapy advances for glioblastoma. Neuro Oncol.

[14] Hamid O, Robert C, Daud A, Hodi FS, Hwu WJ, Kefford R, Wolchok JD, Hersey P, Joseph RW, Weber JS, Dronca R, Gangadhar TC, Patnaik A (2013). Safety and tumor responses with lambrolizumab (anti-PD-1) in melanoma. N Engl J Med.

[15] Robert C, Ribas A, Wolchok JD, Hodi FS, Hamid O, Kefford R, Weber JS, Joshua AM, Hwu WJ, Gangadhar TC, Patnaik A, Dronca R, Zarour H (2014). Anti-programmed-death-receptor-1 treatment with pembrolizumab in ipilimumab-refractory advanced melanoma: a randomised dose-comparison cohort of a phase 1 trial. Lancet.

[16] Powles T, Eder JP, Fine GD, Braiteh FS, Loriot Y, Cruz C, Bellmunt J, Burris HA, Petrylak DP, Teng SL, Shen X, Boyd Z, Hegde PS (2014). MPDL3280A (anti-PD-L1) treatment leads to clinical activity in metastatic bladder cancer. Nature.

[17] Brahmer JR, Tykodi SS, Chow LQ, Hwu WJ, Topalian SL, Hwu P, Drake CG, Camacho LH, Kauh J, Odunsi K, Pitot HC, Hamid O, Bhatia S (2012). Safety and activity of anti-PD-L1 antibody in patients with advanced cancer. N Engl J Med.

[18] Ansell SM, Lesokhin AM, Borrello I, Halwani A, Scott EC, Gutierrez M, Schuster SJ, Millenson MM, Cattry D, Freeman GJ, Rodig SJ, Chapuy B, Ligon AH (2015). PD-1 blockade with nivolumab in relapsed or refractory Hodgkin's lymphoma. N Engl J Med.

[19] Dong H, Strome SE, Salomao DR, Tamura H, Hirano F, Flies DB, Roche PC, Lu J, Zhu G, Tamada K, Lennon VA, Celis E, Chen L (2002). Tumor-associated B7-H1 promotes T-cell apoptosis: a potential mechanism of immune evasion. Nat Med.

[20] Blank C, Brown I, Peterson AC, Spiotto M, Iwai Y, Honjo T, Gajewski TF (2004). PD-L1/B7H-1 inhibits the effector phase of tumor rejection by T cell receptor (TCR) transgenic CD8+ T cells. Cancer Res.

[21] Topalian SL, Hodi FS, Brahmer JR, Gettinger SN, Smith DC, McDermott DF, Powderly JD, Carvajal RD, Sosman JA, Atkins MB, Leming PD, Spigel DR, Antonia SJ (2012). Safety, activity, and immune correlates of anti-PD-1 antibody in cancer. N Engl J Med.

[22] Tumeh PC, Harview CL, Yearley JH, Shintaku IP, Taylor EJ, Robert L, Chmielowski B, Spasic M, Henry G, Ciobanu V, West AN, Carmona M, Kivork C (2014). PD-1 blockade induces responses by inhibiting adaptive immune resistance. Nature.

[23] Madore J, Vilain RE, Menzies AM, Kakavand H, Wilmott JS, Hyman J, Yearley JH, Kefford RF, Thompson JF, Long GV, Hersey P, Scolyer RA (2015). PD-L1 expression in melanoma shows marked heterogeneity within and between patients: implications for anti-PD-1/PD-L1 clinical trials. Pigment Cell Melanoma Res.

[24] Sunshine J, Taube JM (2015). PD-1/PD-L1 inhibitors. Curr Opin Pharmacol.

[25] Topalian SL, Taube JM, Anders RA, Pardoll DM (2016). Mechanism-driven biomarkers to guide immune checkpoint blockade in cancer therapy. Nat Rev Cancer.

[26] Berghoff AS, Kiesel B, Widhalm G, Rajky O, Ricken G, Wohrer A, Dieckmann K, Filipits M, Brandstetter A, Weller M, Kurscheid S, Hegi ME, Zielinski CC (2015). Programmed death ligand 1 expression and tumor-infiltrating lymphocytes in glioblastoma. Neuro Oncol.

[27] Dutoit V, Migliorini D, Dietrich PY, Walker PR (2016). Immunotherapy of malignant tumors in the brain: how different from other sites?. Front Oncol.

[28] Wu G, Diaz AK, Paugh BS, Rankin SL, Ju B, Li Y, Zhu X, Qu C, Chen X, Zhang J, Easton J, Edmonson M, Ma X (2014). The genomic landscape of diffuse intrinsic pontine glioma and pediatric non-brainstem high-grade glioma. Nat Genet.

[29] Yan H, Parsons DW, Jin G, McLendon R, Rasheed BA, Yuan W, Kos I, Batinic-Haberle I, Jones S, Riggins GJ, Friedman H, Friedman A, Reardon D (2009). IDH1 and IDH2 mutations in gliomas. N Engl J Med.

[30] Louis DN, Perry A, Reifenberger G, von Deimling A, Figarella-Branger D, Cavenee WK, Ohgaki H, Wiestler OD, Kleihues P, Ellison DW (2016). The 2016 World Health Organization classification of tumors of the central nervous system: a summary. Acta Neuropathol.

[31] Yamasaki F, Kurisu K, Kajiwara Y, Watanabe Y, Takayasu T, Akiyama Y, Saito T, Hanaya R, Sugiyama K (2011). Magnetic resonance spectroscopic detection of lactate is predictive of a poor prognosis in patients with diffuse intrinsic pontine glioma. Neuro Oncol.

[32] Lachi PK, Irrakula M, Ahmed SF, Joseph D, Pamidighantam S, Jagannath Rao Naidu KV (2015). Clinical profile and outcomes in brainstem glioma: an institutional experience. Asian J Neurosurg.

[33] Reitman ZJ (2014). Smaller protein, larger therapeutic potential: PPM1D as a new therapeutic target in brainstem glioma. Pharmacogenomics.

[34] Rizzo D, Scalzone M, Ruggiero A, Maurizi P, Attina G, Mastrangelo S, Lazzareschi I, Ridola V, Colosimo C, Caldarelli M, Balducci M, Riccardi R (2015). Temozolomide in the treatment of newly diagnosed diffuse brainstem glioma in children: a broken promise?. J Chemother.

[35] Chiang KL, Chang KP, Lee YY, Huang PI, Hsu TR, Chen YW, Chang FC, Wong TT (2010). Role of temozolomide in the treatment of newly diagnosed diffuse brainstem glioma in children: experience at a single institution. Childs Nerv Syst.

[36] Sharma P, Allison JP (2015). The future of immune checkpoint therapy. Science.

[37] Yao Y, Tao R, Wang X, Wang Y, Mao Y, Zhou LF (2009). B7-H1 is correlated with malignancy-grade gliomas but is not expressed exclusively on tumor stem-like cells. Neuro Oncol.

[38] Han S, Zhang C, Li Q, Dong J, Liu Y, Huang Y, Jiang T, Wu A (2014). Tumour-infiltrating CD4(+) and CD8(+) lymphocytes as predictors of clinical outcome in glioma. Br J Cancer.

[39] Kim YH, Jung TY, Jung S, Jang WY, Moon KS, Kim IY, Lee MC, Lee JJ (2012). Tumour-infiltrating T-cell subpopulations in glioblastomas. Br J Neurosurg.

[40] Durand-Panteix S, Farhat M, Youlyouz-Marfak I, Rouaud P, Ouk-Martin C, David A, Faumont N, Feuillard J, Jayat-Vignoles C (2012). B7-H1, which represses EBV-immortalized B cell killing by autologous T and NK cells, is oppositely regulated by c-Myc and EBV latency III program at both mRNA and secretory lysosome levels. J Immunol.

[41] Dahlin AM, Henriksson ML, Van Guelpen B, Stenling R, Oberg A, Rutegard J, Palmqvist R (2011). Colorectal cancer prognosis depends on T-cell infiltration and molecular characteristics of the tumor. Mod Pathol.

[42] Hu S, Li J, Xu F, Mei S, Le Duff Y, Yin L, Pang X, Cen S, Jin Q, Liang C, Guo F (2015). SAMHD1 inhibits LINE-1 retrotransposition by promoting stress granule formation. PLoS Genet.

