# Supplementary material for: Genetic and immune features of resectable malignant brainstem gliomas
Source: Oncotarget. 2017 Jul 28;8(47):82571–82. doi: 10.18632/oncotarget.19653 (PMC5669911; doi:10.18632/oncotarget.19653)
Supplement: Supplementary file 1 [file oncotarget-08-82571-s001.pdf]

Genetic and immune features of resectable malignant brainstem gliomas

SUPPLEMENTARY MATERIALS

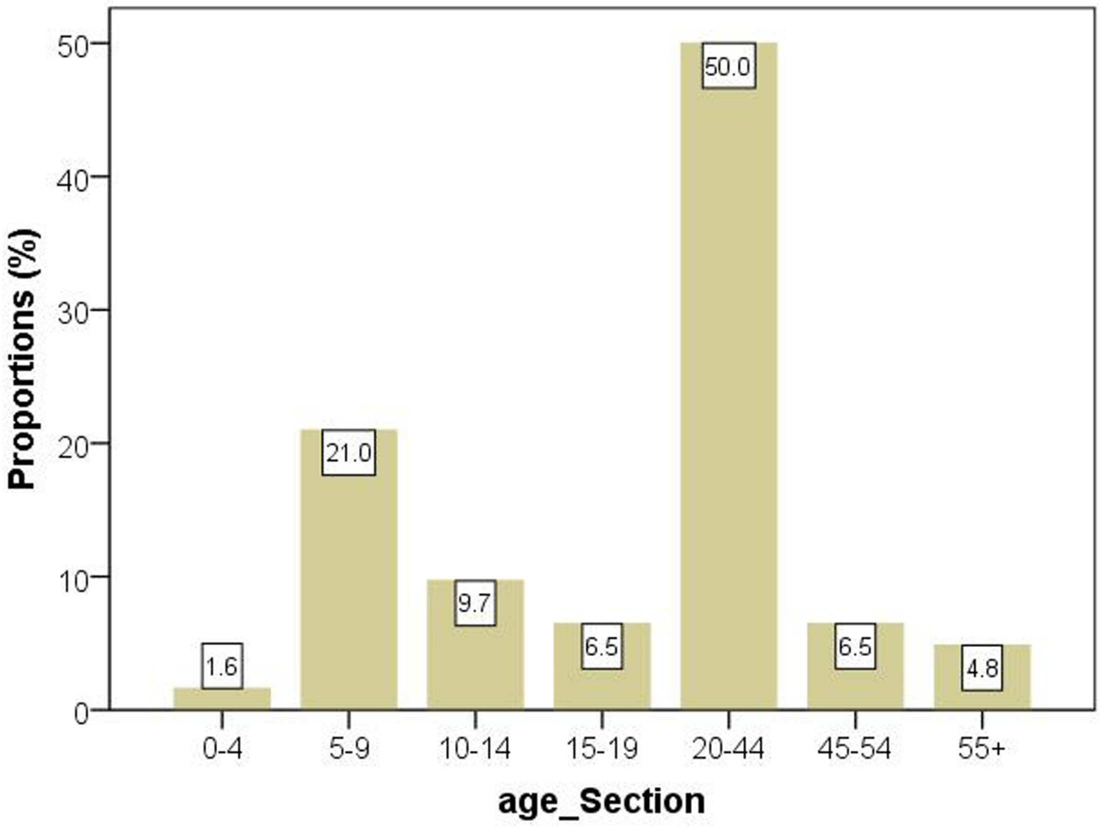

Supplementary Figure 1: Shows the age distribution of the included patients.

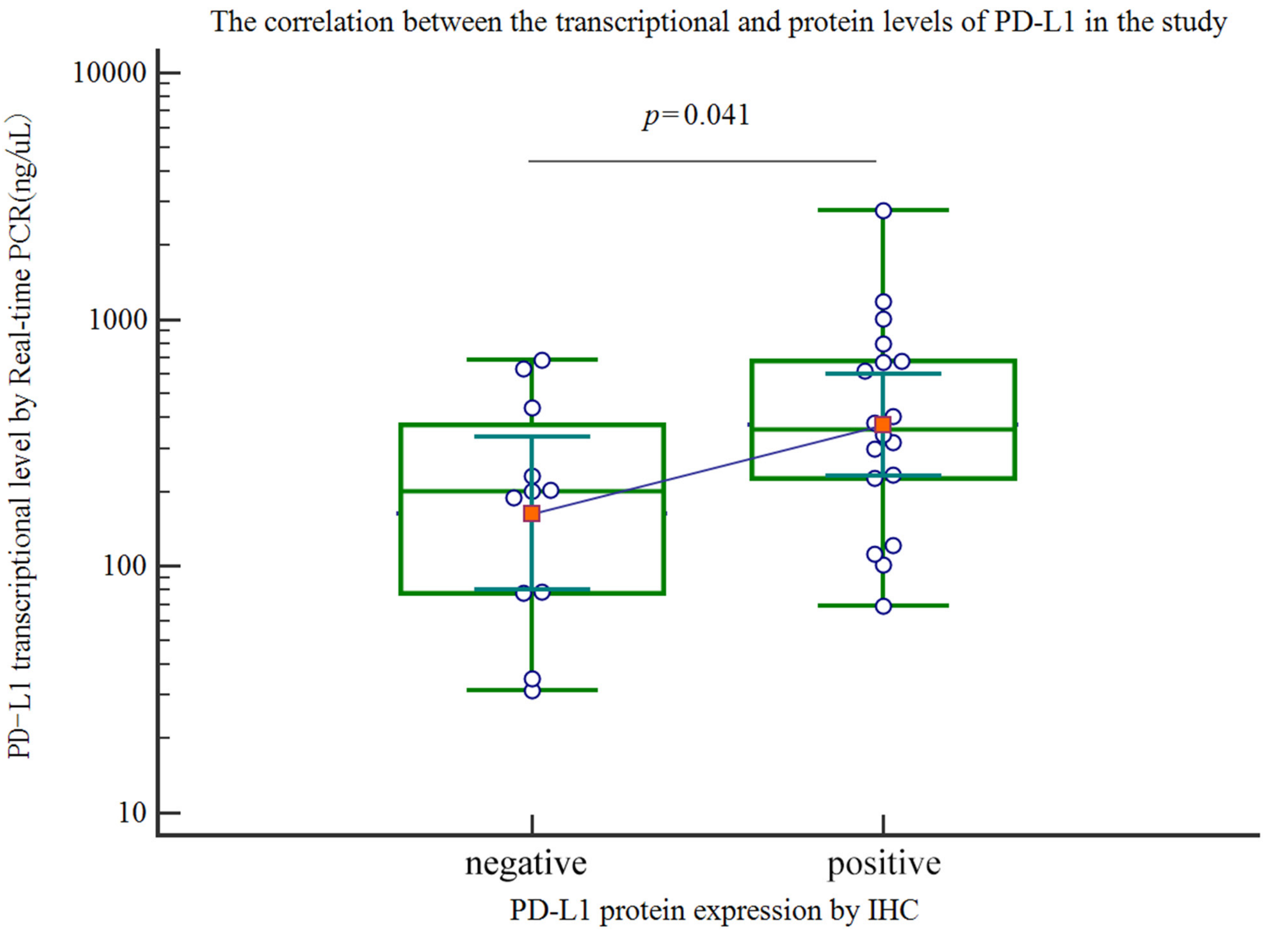

Supplementary Figure 2: Shows that transcriptional level of PD-L1 was significantly higher in the positive PD-L1 staining group than the negative PD-L1 staining, which was detected by the IHC method.

Supplementary Table 1: Distribution of genetic features of resectable brainstem gliomas in patients at different ages

Genetic features		Children and adolescents (0-19 years)				Adults (≥ 20 years)		
		0-4	5-9	10-14	15-19	20-44	45-54	55+
**IDH1/2**	**MUT**	0	0	0	0	7	2	0
	**WT**	1	13	6	4	24	2	3
**H3.3**	**MUT**	0	10	5	4	16	1	1
	**WT**	1	3	1	0	15	3	2
**PPM1D**	**MUT**	0	3	1	1	7	1	0
	**WT**	1	10	5	3	24	3	3
**TP53**	**MUT**	0	6	4	3	13	2	0
	**WT**	1	7	2	1	18	2	3
**MGMT**	**M**	0	0	0	0	5	1	1
	**UM**	1	13	6	4	25	3	2
